# A pap-smear analysis tool (PAT) for detection of cervical cancer from pap-smear images

**DOI:** 10.1186/s12938-019-0634-5

**Published:** 2019-02-12

**Authors:** Wasswa William, Andrew Ware, Annabella Habinka Basaza-Ejiri, Johnes Obungoloch

**Affiliations:** 1 Department of Biomedical Sciences and Engineering, Mbarara University of Science and Technology, Mbarara, 1410 Uganda; 2 Faculty of Computing, Engineering and Science, University of South Wales, Prifysgol, Pontypridd, UK; 3 College of Computing and Engineering, St. Augustine International University, Kampala, Uganda

**Keywords:** Pap-smear, Fuzzy C-means, Cervical cancer

## Abstract

**Background:**

Cervical cancer is preventable if effective screening measures are in place. Pap-smear is the commonest technique used for early screening and diagnosis of cervical cancer. However, the manual analysis of the pap-smears is error prone due to human mistake, moreover, the process is tedious and time-consuming. Hence, it is beneficial to develop a computer-assisted diagnosis tool to make the pap-smear test more accurate and reliable. This paper describes the development of a tool for automated diagnosis and classification of cervical cancer from pap-smear images.

**Method:**

Scene segmentation was achieved through a Trainable Weka Segmentation classifier and a sequential elimination approach was used for debris rejection. Feature selection was achieved using simulated annealing integrated with a wrapper filter, while classification was achieved using a fuzzy C-means algorithm.

**Results:**

The evaluation of the classifier was carried out on three different datasets (single cell images, multiple cell images and pap-smear slide images from a pathology lab). Overall classification accuracy, sensitivity and specificity of ‘98.88%, 99.28% and 97.47%’, ‘97.64%, 98.08% and 97.16%’ and ‘95.00%, 100% and 90.00%’ were obtained for each dataset, respectively. The higher accuracy and sensitivity of the classifier was attributed to the robustness of the feature selection method that accurately selected cell features that improved the classification performance and the number of clusters used during defuzzification and classification. Results show that the method outperforms many of the existing algorithms in sensitivity (99.28%), specificity (97.47%), and accuracy (98.88%) when applied to the Herlev benchmark pap-smear dataset. False negative rate, false positive rate and classification error of 0.00%, 10.00% and 5.00%, respectively were obtained when applied to pap-smear slides from a pathology lab.

**Conclusions:**

The major contribution of this tool in a cervical cancer screening workflow is that it reduces on the time required by the cytotechnician to screen very many pap-smears by eliminating the obvious normal ones, hence more time can be put on the suspicious slides. The proposed system has the capability of analyzing a full pap-smear slide within 3 min as opposed to the 5–10 min per slide in the manual analysis. The tool presented in this paper is applicable to many pap-smear analysis systems but is particularly pertinent to low-cost systems that should be of significant benefit to developing economies.

## Introduction

Cervical cancer is one of the most deadly and common forms of cancer among women in the world [1]. Over 85% of cervical cancer cases occur in less developed countries of which the highest incidences are in Africa, with Uganda being ranked 7th among the countries with the highest incidences of cervical cancer. Over 85% of those diagnosed with the disease in Uganda die from it [2]. This is attributed to lack of awareness of the disease and limited access to screening and health services. Cervical cancer can be prevented if effective screening programmes are in place and this can lead to reduced morbidity and mortality [3]. The success of screening has been reported to depend on a number of factors including access to facilities, quality of screening tests, adequacy of follow-up, diagnosis and treatment of lesions detected [4]. Cervical cancer screening services are very low in low middle-income countries due to the presence of only a few trained and skilled health workers, and the lack of healthcare resources to sustain screening programmes [5]. This is even lower in the East African region where cervical cancer age-standardized incidence rates are highest due to inadequate screening programs [6]. The incidence of cervical cancer can be reduced by regular screening based on the pap-smear test. However, the manual analysis of the pap-smear images is time-consuming, laborious and error-prone as hundreds of sub-images within a single slide have to be examined under a microscope by a trained cytopathologist for each patient during screening. Human visual grading of microscopic images tends to be subjective and inconsistent [7]. Hence, there have been numerous attempts to automate the analysis of pap-smears since its introduction more than 70 years ago [8–10].

### Computer-assisted pap-smear analysis

Since the 1960’s numerous projects have developed computer-assisted pap-smear analysis systems leading to a number of commercial products such as AutoPap 300 [11] and the PapNet [12] which were approved by the United States Food and Drug Administration (FDA) in 1998. A number of other projects have attempted to automate the pap-smear analysis. The Cytoanalyzer developed in the US was the first attempt at building an automated screening device for pap-smears based on the concept of nuclear size and optical density [13]. Unfortunately, tests with the Cytoanalyzer revealed that the device produced too many false rates on the cell level. The CYBEST developed in Japan was based on nucleus area, nucleus density, cytoplasmic area, and nuclear to cytoplasmic ratio [14]. The prototype was used in large field trials in the Japanese screening program and showed promising results but it never became a commercial product. The BioPEPR project was a general image analysis system for cervical cancer screening based on nuclear area, nuclear optical density, nuclear texture, and nuclear to cytoplasmic ratio [15]. There was no in-depth study made to assess the efficiency of BioPEPR system in detecting abnormalities and hence the product did not go to market. Another system that was developed was FAZYTAN [16], based on TV-image pickup and parallel processing. The system was efficient and fast in detection and segmentation of cells scanned in one TV frame within one second as well as the extraction of a large number of morphologic features within a few seconds. FAZYTAN never reached the market, and an important reason for this was lack of cost-effectiveness. In 2007, Cytyc was successful with their improved liquid based preparation technique and received FDA approval for their ThinPrep Imaging System [17]. In 2004, BDFocalPoint Slide Profiler imaging system was developed based on the AutoPap 300 system. However, a new liquid-based specimen preparation technique called SurePath was added to further improve the system performance although it can also analyze conventional pap-smear slides [18]. Despite the availability of these commercial automated cervical cancer screening systems, they have had little impact in low middle-income countries due to the high costs involved in buying and maintaining them [8].

In literature, a number of techniques for automated/semi-automated diagnosis and classification of cervical cancer from pap-smear images have been developed by several researchers as shown in Table 1.

**Table 1:** Some of the available techniques in the literature for automated/semi-automated detection of cervical cancer from pap-smear images

Author	Paper	Datasets	Features	Pre-processing	Segmentation	Classification	Results
Su et al. [19]	Automatic detection of cervical cancer cells by a two-level cascade classification system	Liquid-based cytology slides	20 Morphological and 8 texture features	Histogram equalization and Median filter	Adaptive threshold	C4.5 and Logical Regression classifiers	Recognition rates of 95.6% achieved
Sharma et al. [20]	Classification of clinical dataset of cervical cancer using KNN	Single cells data sets from Fortis Hospital, India	7 morphological features	*Gaussian filter* and histogram equalization	Min–max and edge detection	K-nearest neighbour	Accuracy of 82.9% with fivefold cross-validation
Kumar et al. [21]	Detection and classification of cancer from microscopic biopsy images using clinically significant features	Histology image dataset (histology DS2828)	125 Nucleus and cytoplasm morphologic features	Contrast limited adaptive histogram equalization	K-means segmentation algorithm	K-NN, fuzzy KNN, SVM and random forest-based classifiers	Accuracy, specificity and sensitivity of 92%, 94% and 81%
Chankong et al. [22]	Automatic cervical cell segmentation and classification in Pap smears	Herlev dataset	Morphological features	Median filter	Patch-based fuzzy C-means and FCM	Fuzzy C-means	Accuracies of 93.78% and 99.27% for 7 and 2-class classifications
Talukdar et al. [23]	Fuzzy clustering based image segmentation of pap smear images of cervical cancer cell using FCM algorithm	Colour image	Morphometric, densitometry, colorimetric and textural feature	Adaptive histogram equalization with Otsu’s method	Chaos theory corresponding to R, G and B value	Pixel-level classification and shape analysis	Preserves the colour of the images and data loss is minimal
Sreedevi et al. [24]	Pap smear image-based detection of cervical cancer,	Herlev dataset	Nucleus features	Colour conversions and contrast enhancement	Iterative thresholding method	Based on the area of the nucleus	A sensitivity of 100% and specificity of 90% achieved
Ampazis et al. [25]	Pap-smear classification using efficient second-order neural network	Herlev University Hospital	20 morphological features	Contrast enhancement	Neural networks	LMAM and OLMAM algorithms	Classification accuracy of 98.86% was obtained

In addition to a recent study by William et al. [10], the reviewed papers in this section indicate that there are still weaknesses in the techniques that result in low accuracy of classification in some classes of cells. Further, most of the developed classifiers are tested on preprocessed images (datasets) using commercially available software such as CHAMP software. There is thus a deficit of evidence that these algorithms will work in clinical settings found in developing countries (where 85% of cervical cancer incidences occur) that lack sufficient trained cytologists and the funds to buy the commercial segmentation software. Furthermore, even though commercial automated pap-smear analysis systems are available for more than 20 years they are too expensive and not cost effective for use in low middle-income countries where the cancer incidences are highest [26]. There is a great need for effective automated screening systems to offer affordable screening in the areas where cervical cancer today has the greatest mortality rate, not the least in Africa.

This paper presents the development of a potent tool for the detection of cervical cancer from pap-smear images using an enhanced fuzzy C-means algorithm. The study has proposed an efficient pixel level classifier for accurate segmentation of the nucleus in pap-smear images using trainable weka segmentation whose applicability in cell segmentation has not been fully explored, yet it can provide an alternative to expensive commercial segmentation tools [27]. Unlike in many of the approaches reviewed which work on pre-processed images, the proposed tool employs a three-phase elimination scheme that sequentially removes debris from the pap-smear if deemed unlikely to be a cervix cell. This approach is beneficial as it allows a lower-dimensional decision to be made at each stage. Simulated annealing coupled with a wrapper filter approach has been used to efficiently select an optimum set of features that do not add noise to a classifier. This approach has been proposed elsewhere [28] but, in this paper, the performance of the feature selection is evaluated using a fitness value evaluated using k-fold cross-validation. Finally, the tool is evaluated based on the hierarchical model of the efficacy of diagnostic imaging systems proposed by Fryback and Thornbury [29].

## Methodology

### Image analysis

**Fig. 1:**
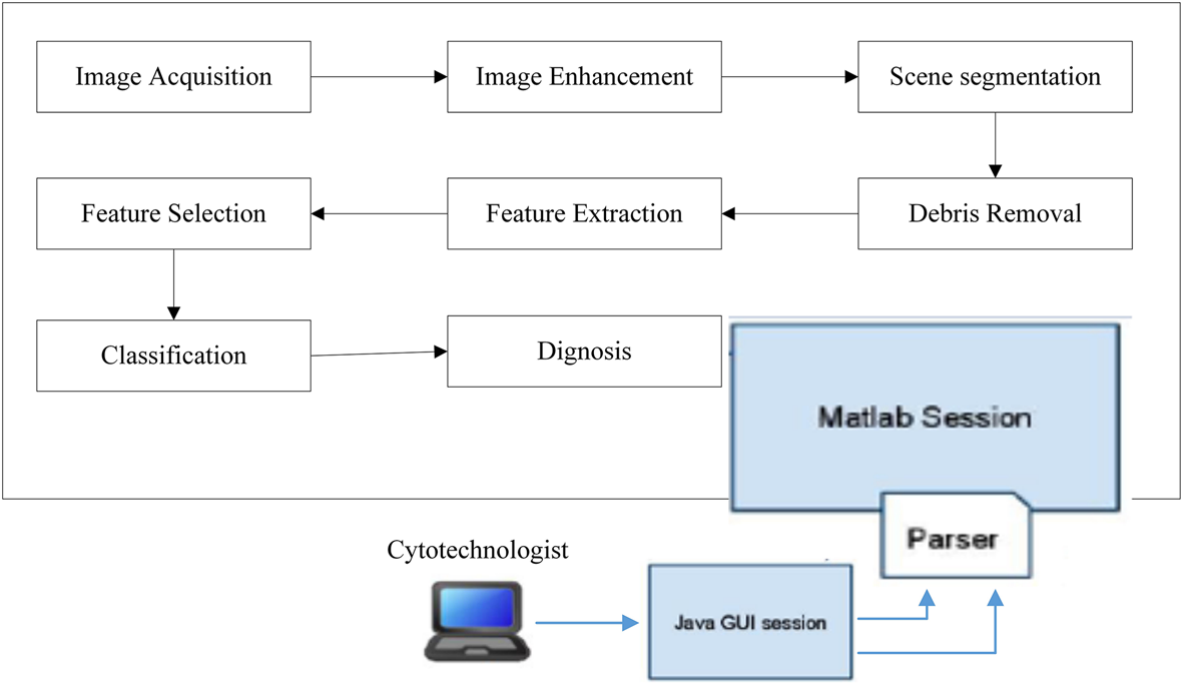
The approach to achieve cervical cancer detection from pap-smear images

The image analysis pipeline for the development of a pap-smear analysis tool for the detection of cervical cancer from pap-smears presented in this paper is depicted in Fig. 1.

#### Image acquisition

The approach was assessed using three datasets. Dataset 1 consists of 917 single cells of Harlev pap-smear images prepared by Jantzen et al. [30]. The dataset contains pap-smear images taken with a resolution of 0.201 µm/pixel by skilled cytopathologists using a microscope connected to a frame grabber. The images were segmented using CHAMP commercial software and then classified into seven classes with distinct characteristics as shown in Table 2. Of these 200 images were used for training and 717 images for testing.

**Table 2:** Some of the characteristics of the cervical cells from the training dataset (N = nucleus, C = cytoplasm)

Cell type	Cancer class	Image	N area	C area	N/C ratio	N bright	C bright	N perimeter	C perimeter
Normal cells	Superficial squamous		631 (±) (206)	61,487 (±) (23,780)	0.01 (±) (0.01)	66 (±) (17)	134 (±) (23)	88 (±) (15)	1034 (±) (221)
	Intermediate squamous		1315 (±) (390)	44,961 (±) (15,345)	0.03 (±) (0.01)	67 (±) (19)	131 (±) (22)	130 (±) (19)	894 (±) (166)
	Columnar epithelial		1591 (±) (699)	3290 (±) (1829)	0.35 (±) (0.10)	94 (±) (25)	138 (±) (36)	153 (±) (35)	323 (±) (103)
Abnormal cells	Mild squamous		4690 (±) (1901)	15,459 (±) (10,539)	0.27 (±) (0.10)	98 (±) (17)	142 (±) (19)	257 (±) (55)	589 (±) (203)
	Moderate squamous		3873 (±) (1651)	7288 (±) (5207)	0.38 (±) (0.12)	92 (±) (15)	135 (±) (18)	231 (±) (49)	443 (±) (141)
	Severe squamous		2949 (±) (1474)	3415 (±) (2276)	0.49 (±) (0.14)	94 (±) (22)	143 (±) (29)	208 (±) (52)	323 (±) (95)
	Carcinoma in situ		2986 (±) (1474)	2115 (±) (1490)	0.60 (±) (0.13)	97 (±) (18)	142 (±) (22)	215 (±) (48)	28 (±) (67)

Dataset 2 consists of 497 full slide pap-smear images prepared by Norup et al. [31]. Of these 200 images were used for training and 297 images for testing. Furthermore, the performance of the classifier was evaluated on Dataset 3 of samples of 60 pap-smears (30 normal and 30 abnormal) obtained from Mbarara Regional Referral Hospital (MRRH). Specimens were imaged using an Olympus BX51 bright-field microscope equipped with a 40×, 0.95 NA lens and a Hamamatsu ORCA-05G 1.4 Mpx monochrome camera, giving a pixel size of 0.25 µm with 8-bit grey depth. Each image was then divided into 300 areas with each area containing between 200 and 400 cells. Based on the opinions of the cytopathologists, 10,000 objects in images derived from the 60 different pap-smear slides were selected of which 8000 were free lying cervical epithelial cells (3000 normal cells from normal smears and 5000 abnormal cells from abnormal smears) and the remaining 2000 were debris objects. This pap-smear segmentation was achieved using Trainable Weka Segmentation toolkit to construct a pixel level segmentation classifier.

#### Image enhancement

A contrast local adaptive histogram equalization (CLAHE) was applied to the grayscale image for image enhancement [32]. In CLAHE, the selection of clip-limit which specifies the desired shape of the histogram of the image is paramount, as it critically influences the quality of the enhanced image. The optimal value of the clip-limit was selected empirically using the method defined by Joseph et al. [33]. An optimum clip limit value of 2.0 was determined to be appropriate for providing adequate image enhancement while preserving the dark features for the datasets used. Conversion to grayscale was achieved using a grayscale technique implemented using Eq. 1 as defined in [34].

1 $$New \;Grayscale \;Image = \left( {\left( {0.3*R} \right) + \left( {0.59*G} \right) + \left( {0.11*B} \right)} \right),$$

where R = Red, G = Green and B = Blue colour contributions to the new image.

Application of CLAHE for image enhancement resulted in noticeable changes to the images by adjusting image intensities where the darkening of the nucleus, as well as the cytoplasm boundaries, became easily identifiable using a clip limit of 2.0.

#### Scene segmentation

To achieve scene segmentation, a pixel level classifier was developed using Trainable Weka Segmentation (TWS) toolkit. The majority of cells observed in a pap-smear are not surprisingly cervical epithelial cells [35]. In addition, varying numbers of leukocytes, erythrocytes and bacteria are usually evident, while small numbers of other contaminating cells and microorganisms are sometimes observed. However, the pap-smear contains four major types of squamous cervical cells—superficial, intermediate, parabasal and basal—of which superficial and intermediate cells represent the overwhelming majority in a conventional smear; hence these two types are usually used for a conventional pap-smear analysis [36]. A trainable Weka segmentation was used to identify and segment the different objects on the slide. At this stage, a pixel level classifier was trained on cell nuclei, cytoplasm, background and debris identification with the help of a skilled cytopathologist using Trainable Weka Segmentation (TWS) toolkit [27]. This was achieved by drawing lines/selection through the areas of interest and assigning them to a particular class. The pixels under the lines/selection were taken to be the representative of the nuclei, cytoplasm, background and debris.

**Fig. 2:**
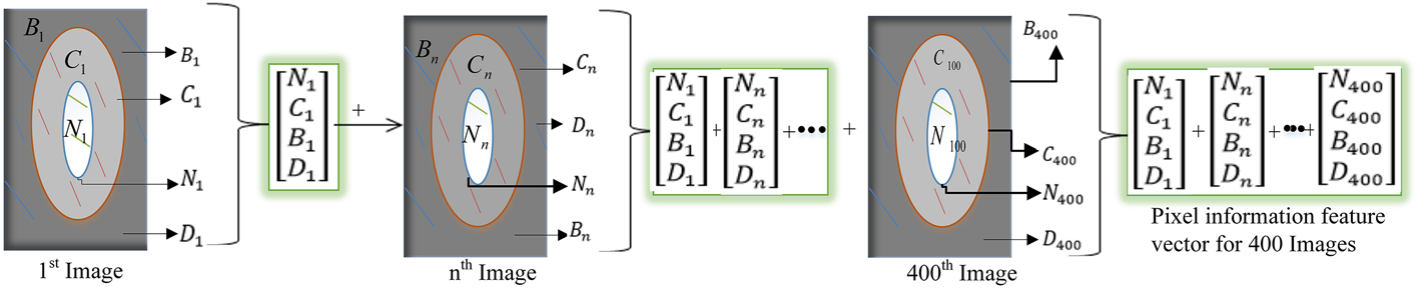
Generation of the feature vector from the training images

The outlines drawn within each class were used to generate a feature vector, $\mathop F\limits^{ \to }$ which was derived from the number of pixels belonging to each outline. The feature vector from each image (200 from Dataset 1 and 200 from Dataset 2) was defined by Eq. 2.

2 $$\vec{F} = \left[ {\begin{array}{*{20}c} {N_{i} } \\ {C_{i} } \\ {B_{i} } \\ {D_{i} } \\ \end{array} } \right],$$

where *N*_*i*_, *C*_*i*_*, B*_*i*_ and *D*_*i*_ are the number of pixels from the nucleus, cytoplasm, background and debris of image $i$ as shown in Fig. 2.

Each pixel extracted from the image represents not only its intensity but also a set of image features that contain a lot of information including texture, borders and colour within a pixel area of 0.201 µm^2^. Choosing an appropriate feature vector for training the classifier was a great challenge and a novel task in the proposed approach. The pixel level classifier was trained using a total of 226 training features from TWS. The classifier was trained using a set of TWS training features which included: (i) Noise Reduction: The Kuwahara [37] and Bilateral filters [38] in the TWS toolkit were used to train the classifier on noise removal. These have been reported to be excellent filters for removing noise whilst preserving the edges [38], (ii) Edge Detection: A Sobel filter [39], Hessian matrix [40] and Gabor filter [41] were used for training the classifier on boundary detection in an image, and (iii) Texture filtering: The mean, variance, median, maximum, minimum and entropy filters were used for texture filtering.

#### Debris removal

**Fig. 3:**
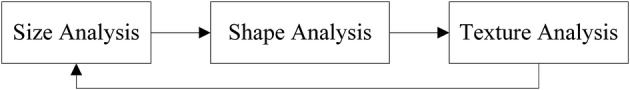
Three-phase sequential elimination approach for debris rejection

The main reason for the current limitations of many of the existing automated pap-smear analysis systems is that they struggle to overcome the complexity of the pap-smear structures, by trying to analyze the slide as a whole, which often contain multiple cells and debris. This has the potential to cause the failure of the algorithm and requires higher computational power [42]. Samples are covered in artefacts—such as blood cells, overlapping and folded cells, and bacteria—that hamper the segmentation processes and generate a large number of suspicious objects. It has been shown that classifiers designed to differentiate between normal cells and pre-cancerous cells usually produce unpredictable results when artefacts exist in the pap-smear [43]. In this tool, a technique to identify cervix cells using a three-phase sequential elimination scheme (depicted in Fig. 3) is used.

The proposed three-phase elimination scheme sequentially removes debris from the pap-smear if deemed unlikely to be a cervix cell. This approach is beneficial as it allows a lower-dimensional decision to be made at each stage.

##### Size analysis

Size analysis is a set of procedures for determining a range of size measurements of particles [44]. The area is one of the most basic features used in the field of automated cytology to separate cells from debris. The pap-smear analysis is a well-studied field with much prior knowledge regarding cell properties [45]. However, one of the key changes with nucleus area assessment is that cancerous cells undergo a substantial increase in nuclear size [43]. Therefore, determining an upper size threshold that does not systematically exclude diagnostic cells is much harder, but has the advantage of reducing the search space. The method presented in this paper is based on a lower size and upper size threshold of the cervical cells. The pseudo code for the approach is shown in Eq. 3.

3 $$If\;Area_{min} \le Area_{roi} \le Area_{max} \;then\;\left\langle {foreground} \right\rangle \;else\;\left\langle {Background} \right\rangle ,$$

where $Area_{max} = 85,267\,{\upmu \text{m}}^{2}$ and $Area_{min} = 625\,{\upmu \text{m}}^{2}$ derived from Table 2.

The objects in the background are regarded as debris and thus discarded from the image. Particles that fall between $Area_{min}$ and $Area_{max}$ are further analysed during the next stages of texture and shape analysis.

##### Shape analysis

The shape of the objects in a pap-smear is a key feature in the differentiation between cells and debris [30]. There are a number of methods for shape description detection and these include region-based and contour-based approaches [46]. Region-based methods are less sensitive to noise but more computationally intensive, whereas contour-based methods are relatively efficient to calculate but more sensitive to noise [43]. In this paper, a region-based method (perimeter2/area (P2A)) has been used [47]. The P2A descriptor was chosen on the merit that it describes the similarity of an object to a circle. This makes it well suited as a cell nucleus descriptor since nuclei are generally circular in their appearance. The P2A is also referred to as shape compactness and is defined by Eq. 4.

4 $$c = \frac{{p^{2} }}{A},$$

where *c* is the value of shape compactness, *A* is the area and *p* is the perimeter of the nucleus. Debris was assumed to be objects with a P2A value greater than 0.97 or less than 0.15 as per the training features (depicted in Table 2).

##### Texture analysis

Texture is a very important characteristic feature that can differentiate between nuclei and debris. Image texture is a set of metrics designed to quantify the perceived texture of an image [48]. Within a pap-smear, the distribution of average nuclear stain intensity is much narrower than the stain intensity variation among debris objects [43]. This fact was used as the basis to remove debris based on their image intensities and colour information using Zernike moments (ZM) [49]. Zernike moments are used for a variety of pattern recognition applications and are known to be robust with regards to noise and to have a good reconstruction power. In this work, the ZM as presented by Malm et al. [43] of order *n* with repetition *I* of function $f\left( {r,\theta } \right)$, in polar coordinates inside a disk centered in square image $I\left( {x,y} \right)$ of size $m \times m$ given by Eq. 5 was used.

5 $$A_{nl} = \frac{n + 1}{\pi }\mathop \sum \limits_{x} \mathop \sum \limits_{y} v_{nl}^{*} \left( {r,\theta } \right)I\left( {x,y} \right),$$

$v_{nl }^{*} \left( {r,\theta } \right)$ denotes the complex conjugate of the Zernike polynomial $v_{nl} \left( {r,\theta } \right)$. To produce a texture measure, magnitudes from $A_{nl}$ centered at each pixel in the texture image are averaged [43].

#### Feature extraction

The success of a classification algorithm greatly depends on the correctness of the features extracted from the image. The cells in the pap-smears in the dataset used are split into seven classes based on characteristics such as size, area, shape and brightness of the nucleus and cytoplasm. The features extracted from the images included morphology features previously used by others [30, 50]. In this paper three geometric features (solidity, compactness and eccentricity) and six textual features (mean, standard deviation, variance, smoothness, energy and entropy) were also extracted from the nucleus, resulting in 29 features in total as shown in Table 3.

**Table 3:** Extracted features from the pap-smear images

	Nucleus		Cytoplasm
1	*Nucleus area* (NA): The actual number of pixels in nucleus. A pixels area is 0.201 µm^2^	16	*Cytoplasm area* (CA): The actual number of pixels inside the nucleus cytoplasm
2	*Nucleus gray level*: The average perceived brightness of the nucleus from Eq. (1)	17	*Cytoplasm gray level*: The average perceived brightness of the cytoplasm. Calculated using Eq. (1)
3	*Nucleus shortest diameter*: The biggest diameter a circle can have when the circle is totally encircled within the nucleus	18	*Cytoplasm shortest diameter:* This is the biggest diameter a circle can have when the circle is totally encircled of the cytoplasm
4	*Nucleus longest diameter:* This is the shortest diameter a circle can have when surrounding the whole nucleus	19	*Cytoplasm longest diameter:* This is the shortest diameter a circle can have when surrounding the whole cytoplasm
5	*Nucleus elongation*: The ratio between the shortest and longest diameter of the nucleus	20	*Cytoplasm elongation:* The ratio between the shortest diameter and the longest diameter of the cytoplasm
6	*Nucleus roundness:* The ratio between the actual area and the area bound by the circle given by the longest diameter of the nucleus	21	*Cytoplasm roundness:* The ratio between the actual area and the area bound by the circle given by the longest diameter of the cytoplasm
7	*Nucleus perimeter:* The length of the perimeter around the nucleus	22	*Cytoplasm perimeter*: The length of the perimeter around the cytoplasm
8	*Maxima in nucleus:* Maximum number of pixels inside of a three-pixel radius of nucleus	23	*Maxima in cytoplasm*: Maximum number of pixels inside of a three-pixel radius of cytoplasm
9	*Minima in nucleus*: Minimum number of pixels inside of a three-pixel radius of nucleus	24	*Minima in cytoplasm*: Minimum number of pixels inside of a three-pixel radius of nucleus
10	*Nucleus to cytoplasm ratio*: The relative size of the nucleus to the cytoplasm. $\frac{NA}{NA + CA}$	25	*Nucleus relative position*: A measure of how well the nucleus is centred in the cytoplasm
11	*Nucleus solidity*: The proportion of the pixels in the convex hull that is also in the nucleus	26	*Nucleus compactness*: The ratio of area and square of the perimeter of the nucleus
12	*Nucleus eccentricity:* The eccentricity of the ellipse that has the same second-moments as the nucleus region	27	*Nucleus mean:* The mean gray values of the nucleus region
13	*Nucleus standard deviation:* The deviation of gray values of the nucleus region	28	*Nucleus smoothness*: The local variation in radius lengths of the nucleus region
14	*Nucleus variance:* The variance value of the gray values inside the nucleus region	29	*Nucleus energy*: The energy of gray values of the nucleus region
15	Nucleus entropy: The entropy of gray values of the nucleus region		

#### Feature selection

Feature selection is the process of selecting subsets of the extracted features that give the best classification results. Among those features extracted, some might contain noise while the chosen classifier may not utilize others. Hence, an optimum set of features has to be determined, possibly by trying all combinations. However, when there are many features, the possible combinations explode in number and this increases the computational complexity of the algorithm. Feature selection algorithms are broadly classified into the filter, wrapper and embedded methods [51].

**Fig. 4:**
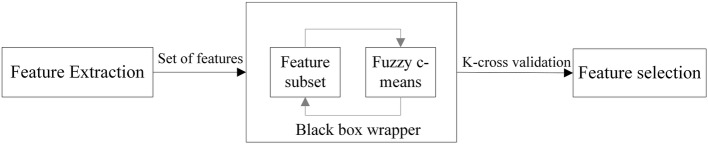
The fuzzy C-means is wrapped into a black box from which an estimated error is obtained

The method used by the tool combines simulated annealing with a wrapper approach. This approach has been proposed in [28] but, in this paper, the performance of the feature selection is evaluated using a double-strategy random forest algorithm [52]. Simulated annealing is a probabilistic technique for approximating the global optimum of a given function. The approach is well suited for ensuring that the optimum set of features is selected. The search for the optimum set is guided by a fitness value [53]. When simulated annealing is finished, all the different subsets of features are compared and the fittest (that is, the one that performs the best) selected. The fitness value search was obtained with a wrapper where k-fold cross-validation was used to calculate the error on the classification algorithm. Different combinations from the extracted features are prepared, evaluated and compared to other combinations. A predictive model is then used to evaluate a combination of features and assign a score based on model accuracy. The fitness error given by the wrapper is used as the fitness error by the simulated annealing algorithm. A fuzzy C-means algorithm was wrapped into a black box, from which an estimated error was obtained for the various feature combinations as shown in Fig. 4.

Fuzzy C-means allows data points in the dataset to belong to all of the clusters, with memberships in the interval (0–1) as shown in Eq. 6.

6 $$m_{ik} = \frac{1}{{\mathop \sum \nolimits_{j = 1}^{c} \left( {\frac{{d_{ik} }}{{d_{jk} }}} \right)^{{2/\left( {q - 1} \right)}} }} ,$$

where $m_{ik}$ is the membership for data point *k* to cluster center *i*, $d_{jk}$ is the distance from cluster center *j* to data point *k* and q €[1…∞] is an exponent that decides how strong the memberships should be. The fuzzy C-means algorithm was implemented using the fuzzy toolbox in Matlab.

#### The defuzzification

A fuzzy C-means algorithm does not tell us what information the clusters contain and how that information shall be used for classification. However, it defines how data points are assigned membership of the different clusters and this fuzzy membership is used to predict the class of a data point [54]. This is overcome through defuzzification. A number of defuzzification methods exist [55–57]. However, in this tool, each cluster has a fuzzy membership (0–1) of all classes in the image. Training data are assigned to the cluster nearest to it. The percentage of training data of each class belonging to cluster *A* gives the cluster’s membership, *cluster A *=* [i, j]* to the different classes, where *i* is the containment in cluster *A* and *j* in the other cluster. The intensity measure is added to the membership function for each cluster using a fuzzy clustering defuzzification algorithm. A popular approach for defuzzification of fuzzy partition is the application of the maximum membership degree principle where data point *k* is assigned to class *m* if, and only if, its membership degree $m_{ik}$ to cluster *i*, is the largest. Chuang et al. [58] proposed adjusting the membership status of every data point using the membership status of its neighbors.

In the proposed approach, a defuzzification method based on Bayesian probability is used to generate a probabilistic model of the membership function for each data point and apply the model to the image to produce the classification information. The probabilistic model [59] is calculated as below:

1. Convert the possibility distributions in the partition matrix (clusters) into probability distributions.
2. Construct a probabilistic model of the data distributions as in [59].
3. Apply the model to produce the classification information for every data point using Eq. 7.

7 $${\text{P}}\left( {A_{i} |B_{j} } \right) = \frac{{P\left( {B_{j} |A_{i} } \right)*P\left( {A_{i} } \right)}}{{B_{j} }} ,$$

where $P\left( {A_{i} } \right),i = 0 \ldots .c$ is the prior probability of $A_{i}$ which can be computed using the method in [59, 60] where the prior probability is always proportional to the mass of each class.

The number of clusters to use was determined to ensure that the built model can describe the data in the best possible way. If too many clusters are chosen, then there is a risk of overfitting the noise in the data. If too few clusters are chosen, then a poor classifier might be the result. Therefore, an analysis of the number of clusters against the cross-validation test error was performed. An optimal number of 25 clusters was attained and overtraining occurred above these number of clusters. A defuzzification exponent of 1.0930 was obtained with 25 clusters, tenfold cross-validation and 60 reruns and was used to calculate the fitness error for feature selection where a total of 18 features out of the 29 features were selected for construction of the classifier. The selected features were: nucleus area; nucleus gray level; nucleus shortest diameter; nucleus longest; nucleus perimeter; maxima in nucleus; minima in nucleus; cytoplasm area; cytoplasm gray level; cytoplasm perimeter; nucleus to cytoplasm ratio; nucleus eccentricity, nucleus standard deviation, nucleus gray level variance; nucleus gray level entropy; nucleus relative position; nucleus gray level mean and nucleus gray values energy.

#### Classification evaluation

In this paper, the hierarchical model of the efficacy of diagnostic imaging systems proposed by Fryback and Thornbury [29] was adopted as a guiding principle for the evaluation of the tool as shown in Table 4.

**Table 4:** Tool evaluation criteria

Diagnostic efficacy	Evaluation metrics
Technical efficacy	How well the tool extracts features used for classification? These included nucleus and cytoplasm areas, perimeters etc.
Diagnostic accuracy efficacy	Classification accuracy, sensitivity, specificity, false positive rate and false negative rate

Sensitivity measures the proportion of actual positives that are correctly identified as such whereas specificity measures the proportion of actual negatives that are correctly identified as such. Sensitivity and specificity are described by Eq. 8.

8 $$Sensitivity \;\left( {TPR} \right) = \frac{TP}{TP + FN},\;Specificity\; \left( {TNR} \right) = \frac{TN}{TN + FP},$$

where TP = True positives, FN = False negatives, TN = True negatives and FP = False positives.

### GUI design and integration

**Fig. 5:**
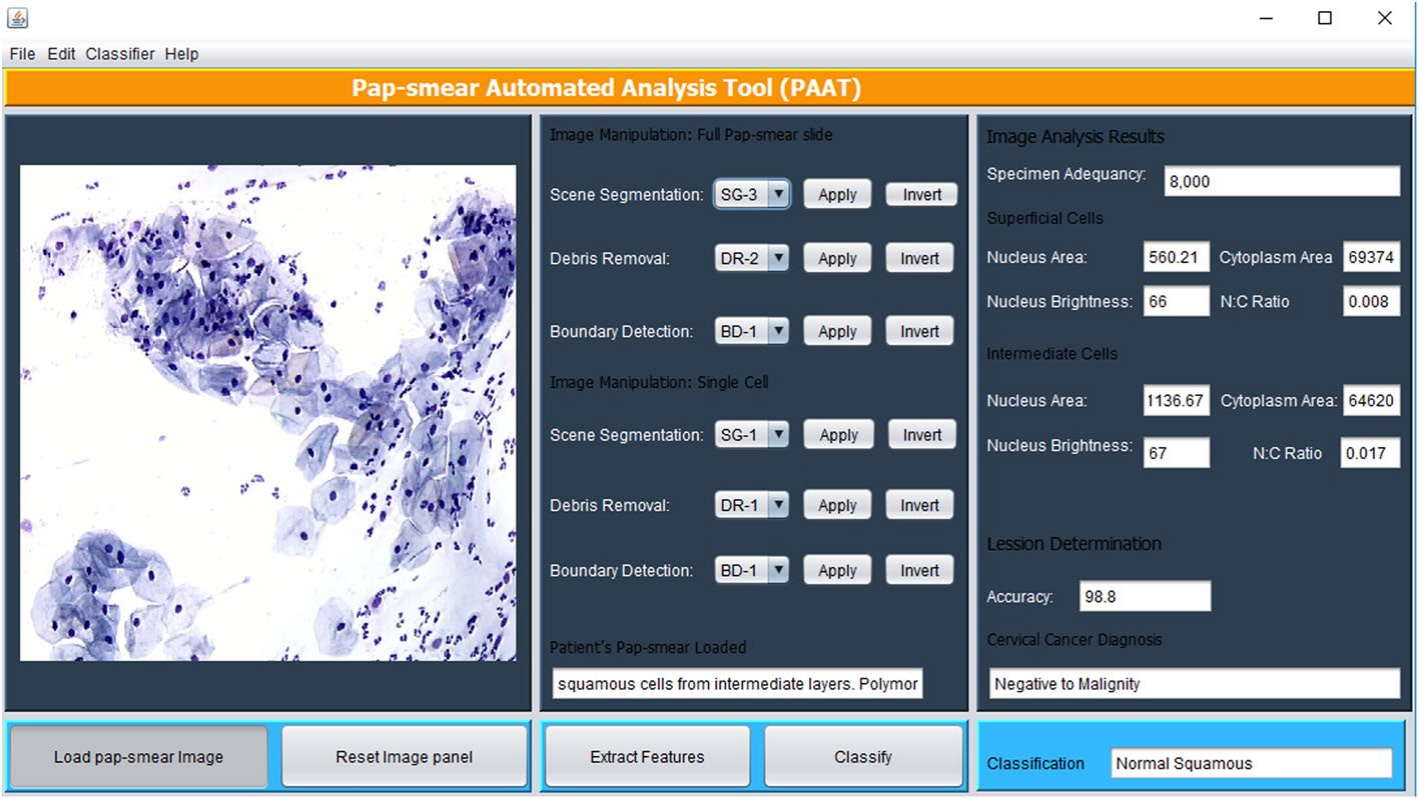
PAT graphical user interface

The image processing methods described above were implemented in Matlab and are executed via a Java graphical user interface (GUI) shown in Fig. 5. The tool has a panel where a pap-smear image is loaded and the cytotechnician selects an appropriate method for scene segmentation (based on TWS classifier), debris removal (based on the three sequential elimination approach) and boundary detection (if deemed necessary, using Canny edge detection method), after which features are extracted using the extract features button.

The tool scans through the pap-smear to analyze all the objects that remained after debris removal. The 18 features described in feature selection are extracted from each object and used to classify each cell using the fuzzy C-means algorithm described in the classification method. Randomly, extracted features of one superficial cell and one intermediate cell are displayed in the image analysis results panel. Once the features have been extracted, the cytotechnician (user) presses the classify button and the tool emits a diagnosis (positive to malignity or negative to malignity) and classifies the diagnosis to one of the 7 classes/stages of cervical cancer as per the training dataset.

## Results

### Technical efficacy

Technical efficacy assessed how well the tool extracted cell features from the segmented images. The tool was used to segment cervical cells as depicted in Table 5.

**Table 5:**
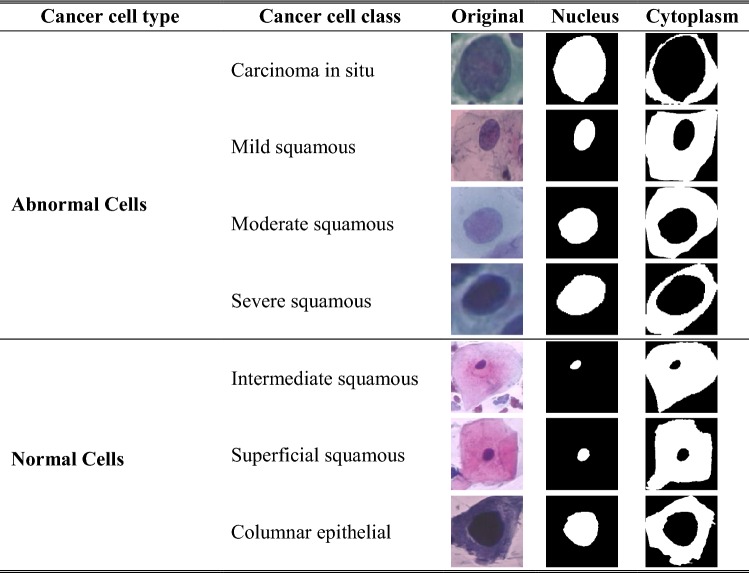
Nucleus and cytoplasm segmentation using the proposed method

Comparison of the segmented nucleus and cytoplasm with the ground truth nucleus and cytoplasm segmentations resulted into average Zijdenbos similarity index (ZSI) of 0.9725 and 0.9483 for the nucleus and cytoplasm segmentation, respectively. The tool was then used to extract features for cervical cancer classification. The features extracted included the nucleus area, nucleus brightness, cytoplasm area and nucleus to cytoplasm ratio. The tool was used to extract cell features from 50 random single cells from the test images from the Herlev dataset (Dataset 1) and compared with the features reported by Martin et al. [61] extracted using CHAMP commercial software. The percentage errors within the measurements for a single test image are shown in Table 6.

**Table 6:** Comparison of the extracted features from a normal superficial cell by CHAMP and PAT

Features	CHAMP	PAT	%|Error|
Nucleus area	562.38 µm^2^	563.64 µm^2^	0.22
Cytoplasm area	69,395.88 µm^2^	69,430.30 µm^2^	0.05
Nucleus brightness	66.00	66.14	0.21
Nucleus to cytoplasm ratio	0.00810	0.00811	0.17

**Fig. 6:**
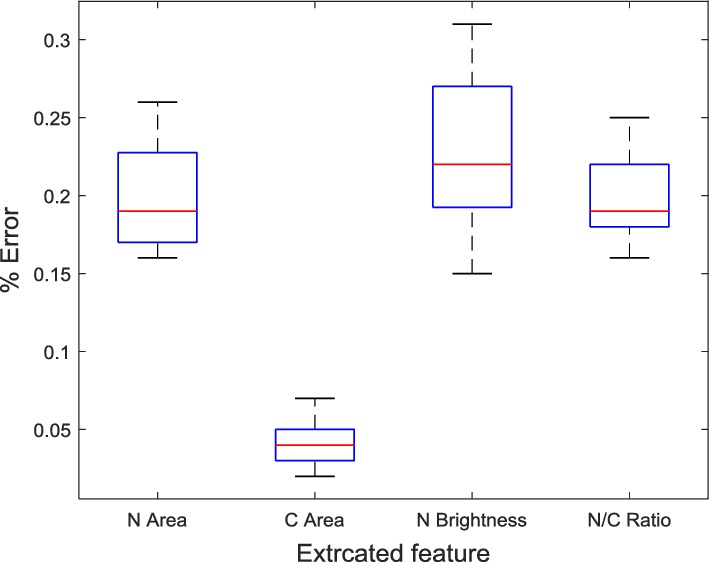
Boxplot for the percentage error in the extracted features

A box plot was obtained to show the shape of the distribution of the percentage error, its central value, and its variability in each of the extracted features for the 50 test cells as shown in Fig. 6.

The tool’s efficacy to extract cell features from a full pap-smear image was also evaluated. The tool was used to extract cell features from a normal cell from 50 normal test pap-smear images obtained from Mbarara Regional Referral Hospital. The pap-smear has many cells but the cell with the highest nucleus area was identified by a cytopathologist. The aim was for PAT to scan through all the cells, extract and evaluate the individual cell features and extract the cell features of the cell with the highest nucleus area. The results are shown in Table 7.

**Table 7:** Comparison of the extracted features from a normal superficial cell by a cytopathologist and PAT

Superficial cell feature	Evaluation		%|Error|
	Cytopathologist	PAT	
Nucleus area	1328 µm^2^	1331.67 µm^2^	0.27
Cytoplasm area	44,991 µm^2^	45,001.85 µm^2^	0.02
Nucleus brightness	67 (light)	67.32	0.41
Nucleus to cytoplasm ratio	0.02951 (Small)	0.02959	0.25

The same features were extracted from cells obtained from 50 abnormal pap-smears and results extracted by the cytopathologist compared with those extracted by PAT. The results of a single cell are presented in Table 8.

**Table 8:** Comparison of the extracted features from an abnormal superficial cell by a cytopathologist and PAT

Superficial cells features	Evaluation		%|Error|
	Cytopathologist	PAT	
Nucleus area	3996 µm^2^	4006.67 µm^2^	0.26
Cytoplasm area	7188 µm^2^	7191.40 µm^2^	0.04
Nucleus brightness	97 (very dark)	97.31	0.31
Nucleus to cytoplasm ratio	0.555 (very large)	0.5571	0.21

**Fig. 7:**
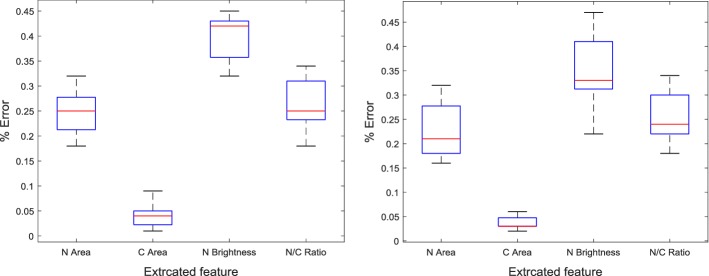
Boxplot for the percentage error in the extracted features from 50 normal pap-smear slides (first boxplot) and 50 abnormal pap-smear slides (second boxplot)

Similarly, to show the shape of the distribution of the percentage error, its central value, and its variability in each of the extracted features from the 50 cells obtained from pap-smears, box plots were obtained as shown in Fig. 7.

### Diagnostic accuracy efficacy

This was used to evaluate the classification accuracy, sensitivity, specificity, false negative rate and the false positive rate on the three sets of datasets. A confusion matrix for the classification results on the test single cells (Dataset 1 consisting of 717 test single cells) is shown in Table 9. Of the 158 normal cells, 154 were correctly classified as normal and four were incorrectly classified as abnormal (one normal superficial, one intermediate and two normal columnar). Of the 559 abnormal cells, 555 were correctly classified as abnormal and four were incorrectly classified as normal (two carcinoma in situ cell, one moderate dysplastic and one mild dysplastic). The overall accuracy, sensitivity and specificity of the classifier on this dataset was 98.88%, 99.28% and 97.47%, respectively. A false negative rate (FNR), false positive rate (FPR) and classification error of 0.72%, 2.53% and 1.12%, respectively were obtained.

**Table 9:** Cervical cancer classification results from single cells

Abnormal		Normal	
False negative	4	True negative	154
True positive	555	False positive	4
Total	559	Total	158

**Fig. 8:**
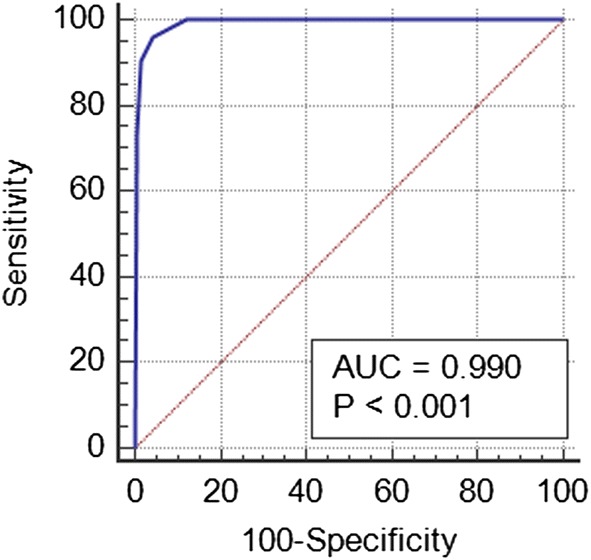
ROC curve for the classifier performance on Dataset 1

A receiver operating characteristic (ROC) curve was plotted to analyze how the classifier can distinguish between the true positives and negatives. This was necessary because the classifier needs to not only correctly predict a positive as a positive, but also a negative as a negative. This ROC was obtained by plotting sensitivity (the probability of predicting a real positive as positive), against 100-specificity (the probability of predicting a real negative as negative) as shown in Fig. 8.

A confusion matrix for the classification results on test pap-smear slides (Dataset 2 of 297 full slide test images) is shown in Table 10. Of the 141 normal slides, 137 were correctly classified as normal and four were incorrectly classified as abnormal. Of the 156 abnormal slides, 153 were correctly classified as abnormal and three were incorrectly classified as normal. The overall accuracy, sensitivity and specificity of the classifier on this dataset was 97.64%, 98.08% and 97.16%, respectively. A false negative rate, false positive rate and classification error of 1.92%, 2.84% and 2.36%, respectively were obtained.

**Table 10:** Cervical cancer classification results from single cells

Abnormal		Normal	
False negative	3	True negative	137
True positive	153	False positive	4
Total	156	Total	141

Furthermore, the tool was evaluated on a dataset of 60 full pap-smear images (Dataset 3 of 30 normal and 30 abnormal pap-smear images) that had been prepared and classified by a cytotechnologist as normal or abnormal at Mbarara Regional Referral Hospital. Of the 30 normal pap-smears, 27 were correctly classified as normal and three were incorrectly classified as abnormal. All the 30 abnormal slides were correctly classified as abnormal. The overall accuracy, sensitivity and specificity of the tool on this dataset was 95.00%, 100% and 90.00%, respectively. A false negative rate, false positive rate and classification error of 0.00%, 10.00% and 5.00%, respectively were obtained as shown in the confusion matrix in Table 11.

**Table 11:** Cervical cancer classification results from pap-smear cells

Abnormal slides		Normal slides	
False negative	0	True negative	27
True positive	30	False positive	3
Total	30	Total	30

The proposed tool’s performance was compared with state of art classification algorithms documented in the relevant literature as shown in Table 12. Results showed that the proposed method outperforms many of the documented algorithms in terms of classification cell level accuracy (98.88%), specificity (97.47%) and sensitivity (99.28%), when applied to the Herlev benchmark pap-smear dataset (single cell dataset).

**Table 12:** Comparison of the developed classifier’s performance with methods in [62–64]

Method	Method	Sensitivity (%)	Specificity (%)	Accuracy (%)
Zhang et al. [62]	Deep convolutional networks	98.2	98.3	98.3
Bora et al. [64]	Ensemble classifier	99.0	89.7	96.5
Marinakis et al. [63]	Genetic algorithm	98.5	92.1	96.8
Proposed Tool (PAT)	Enhanced Fuzzy C-means	99.28	97.47	98.88

### Processing time analysis

The tool was tested on an Intel Core i5-6200U CPU@2.30 GHz 8 GB memory computer. Twenty randomly selected full pap-smear images were run through the algorithm and the computational time measured for both the individual steps and overall duration. Overall time taken per pap-smear image averaged 161 s, and was three minutes at most, demonstrating the feasibility for real-time diagnosis of the pap-smear as opposed to the testing time of 3.5 s for one cervical cell by the method in [62].

## Discussion

A Trainable Weka Segmentation was utilized to provide a cheaper alternative to tools such as CHAMP for scene segmentation. The constructed pixel level classifier produced excellent segmentations for the single images as shown in Table 5. However, segmentation results from full slide pap-smear images required more pre-processing before feature extraction. TWS has been used in many studies and its accuracy is largely dependent on the accuracy of training the pixel level classifier [27, 65, 66]. Increasing the training sample as reported by Maiora et al. [67] could improve the performance of the classifier. TWS’s capability to produce good segmentation is due to its pixel level classification where each pixel is assigned to a given class. However, the poor performance to segment the whole slide would be attributed to the small dataset used for building the segmentation classifier, as this was a manual process that involved annotation by an experienced cytopathologist. Feature selection played an important role in this work since it eliminated features that increased error in the classification algorithm. Eighteen out of the twenty-nine extracted features were selected for classification purpose. It was noted that most of the features that added noise to the classifier were cytoplasmic features. This could be attributed to the difficulty in separating the cytoplasm from the background as opposed to the nucleus, which is darker [68]. Increasing the number of clusters during feature selection reduced the fuzziness exponent. This implies that increasing the number of clusters reduces the defuzzification error computed by the defuzzification method presented in this paper, which is based on Bayesian probability to generate a probabilistic model of the membership function for each data point and apply the model to the image to produce the classification information. An optimal number of 25 clusters was attained and overtraining occurred when too many clusters (above 25) were used. This is due to the defuzzification method used whose density measure works against overfitting by giving smaller clusters less influence than larger clusters. The overall accuracy of the tool could be attributed to the fuzzy membership that is assigned to each class, and the relevance of the nucleus features selected.

The results in Table 6 show that the proposed tool can extract similar features as those extracted by commercially available expensive CHAMP software. The results in Tables 7 and 8 show that the feature measurements obtained by the proposed tool are in agreement with those obtained by the cytotechnician. Detection of cervical cancer cells is dependent on a number of morphological cell features; hence it is likely that the tool and the cytopathologist will emit a similar diagnosis on the same image. This is also shown by the least variations in the percentage errors in the extracted feature shown in the boxplots in Figs. 6, 7.

The results in Table 9 are representative of the results that can be obtained from single cells, hence they provide a lower limit for the false negative and false positive rates on the cell level of 0.72% and 2.53%, respectively. This implies that if the classifier is presented with well-prepared slides then higher sensitivity values (> 99%) can always be obtained, as seen from the ROC curve in Fig. 8. The results in Table 10 are representative of the results that can be obtained from pre-processed full slide smears. False negative and false positive rates on the smear level of 1.92% and 2.84%, respectively were obtained. This implies that if the tool is presented with well-prepared slides then higher sensitivity values can always be obtained. The tool again showed excellent results in the classification of a pap-smear slide as cancerous with a sensitivity of 98.08%. The results in Table 11 are representative of the results that can be obtained from a pap-smear slide from the pathology laboratory. A smear level false negative rate of 0.00% means that no abnormal cells were classified as normal, and therefore, the misclassification of an abnormal smear is unlikely. However, the 10.00% false positive rate means that some normal cells were classified as abnormal. However, confirmation tests are required to be carried out by the cytopathologist. The overall accuracy, sensitivity and specificity of the classifier on full pap-smear slides from the pathology lab was 95.00%, 100% and 90.00% respectively. The higher sensitivity of the tool to cancerous cells could be attributed to the robustness of the feature selection method that selected strict nucleus constrained features that potentially indicate signs of malignancy. Despite the high performance of the approach, it, however, uses numerous methods which makes it computationally expensive and this is a limitation of the proposed method. In the future, deep learning approaches will be explored to reduce the complexity of the approach and also carry out more testing of the tool with more datasets.

## Conclusion

In this paper, we have presented a pap-smear analysis tool for detection of cervical cancer from pap-smear images. The major contribution of this tool in a cervical cancer screening workflows is that it reduces on the time required by the cytotechnician to screen very many pap-smears by eliminating the obvious normal ones, hence more time can be put on the suspicious slides. Normally, a conventional pap-smear slide of size (5.7 × 2.5) mm obtained using a multi-head Olympia microscope may contain around 5000–12,000 cells and it may take 5–10 min for manual analysis. The proposed tool has the capability of analyzing the full pap-smear slide within 3 min. With increased computer speed, efficiently written programs and implementation of this project using Deep learning has the potential to reduce the processing time with more reliable results. The evaluation and testing conducted with the Herlev database and pap-smear slides from Mbarara Regional Referral Hospital prove the validity of the tool and achieving its aim of identifying the cancerous slides/cells that may need more attention. In the future work, we plan to include a cervical cancer risk factors assessment into the tool.

## Abbreviations

WEKA: Waikato Environment for Knowledge Analysis
TWS: trainable weka segmentation
FRF: fast random forest
FCM: fuzzy C-means
